# Sliding Rail Technique (Mandelli's), a Better Method to Repair Thoracoabdominal Aortic Aneurysms Controlled Release Fenestrated Endoprosthesis

**DOI:** 10.1055/a-2541-8455

**Published:** 2025-04-22

**Authors:** Nilo César Barbosa Mandelli, Felipe Figueiró Teixeira, Felipe do Couto Soares de Paula Barros, Laura Vicentini Correa Brunstein

**Affiliations:** 1 Department of Vascular and Endovascular Surgery, IC-FUC: Instituto de Cardiologia, Porto Alegre, Rio Grande do Sul, Brazil; 2 Department of Vascular Surgery, Hospital Universitário Clementino Fraga Filho, Universidade Federal do Rio de Janeiro, Rio de Janeiro, Brazil; 3 Universidade Luterana do Brasil (ULBRA), Canoas, Rio Grande do Sul, Brazil

**Keywords:** FEVAR, TAAAs, juxtarenal aneurysms, double brachial access, controlled-release fenestrated endoprosthesis

## Abstract

Fenestrated endovascular aortic aneurysm repair is a minimally invasive technique used for the treatment of thoracoabdominal aortic aneurysms (TAAAs). We report an easy method for positioning the controlled-release fenestrated endoprosthesis, associated with a less invasive approach for positioning the endoprosthesis and catheterization of the target vessels through percutaneous access.

Fenestrated endovascular aortic aneurysm repair (FEVAR) is a minimally invasive technique widely used for the treatment of thoracoabdominal aortic aneurysms (TAAAs), especially in cases without an adequate neck below the visceral arteries. An example of this is juxtarenal aneurysms, in which there are short necks, with less than 20 mm of fixation. These have a 9 to 10% chance of needing reintervention within 5 years, if the prostheses are placed infrarenal, since there is degeneration of the aortic wall at the site. 1

FEVAR has been shown to be effective in reducing the incidence of renal injury, in addition to decreasing the morbidity and mortality associated with treatment. However, this technique has a higher reintervention rate compared with open surgery, which is still considered the gold standard for treatment. 2 In emergency situations, the use of stents in parallel, such as the “chimney” or “snorkel” techniques, remains a viable alternative, although the technical result may be compromised when more than two vessels need to be incorporated, due to the higher rates of endoleaks. 3

However, when performed by experienced surgeons, FEVAR demonstrates a high technical success rate, with reported success reaching 100% in some cohorts and a perioperative mortality rate as low as 1.5% within 30 days. 1 Furthermore, 1-year tomographic follow-up demonstrates good patency of the fenestrated vessels freedom from aortic-related death at 3 years is high. 4

The use of only one femoral artery for working access favors patient recovery, since, by keeping the contralateral femoral artery free, limb ischemia, and the systemic inflammatory response are reduced. 5 However, catheterization of the visceral branches through the upper limbs, through the brachial artery, is still controversial. Risks of cerebrovascular ischemic events are 1.7% through the left upper limb, 4% through the right upper limb, and 5% through bilateral access, as reported in a systematic review and high-reliability meta-analysis. 6

Despite these concerns, studies indicate that brachial access may be a viable alternative when femoral access is not possible or preferred, especially in complex cases. With careful patient selection and an experienced surgical team, brachial access can minimize the associated risks, providing greater control and facilitating catheterization of the visceral branches, making this a safe and effective option, comparable to femoral access. 7

## Technique

The Mandelli technique was developed to facilitate the implantation of the aortic endoprosthesis in a different location than that recommended in traditional techniques. Traditionally, the target artery needs to be treated together with the endoprosthesis, in its exact original position, since it is not possible to move it once it is opened. This makes for difficult execution. This new technique allows the surgeon to work outside the area of origin of the target artery, facilitating work with the endoprosthesis in a straight and healthy segment of the aorta, whether in the thorax or even in the abdominal aorta (in its healthiest area). This promotes visualization of the correct angle of entry and catheterization of the target arteries, even in aneurysms with a hostile neck.

Furthermore, access can be made through the brachial artery (unilateral or bilateral), axillary artery (unilateral or bilateral), or, if reflex sheaths are used, access through the contralateral femoral artery. In cases of type 3 aortic arch, a larger caliber sheath should be used, such as 12 French × 45 cm. Inside it, another 6 French or 8 French × 90 cm sheath is used; the 12 French then becomes a support for the arch, with the aim of reducing cerebral embolism.

**Fig. 1:**
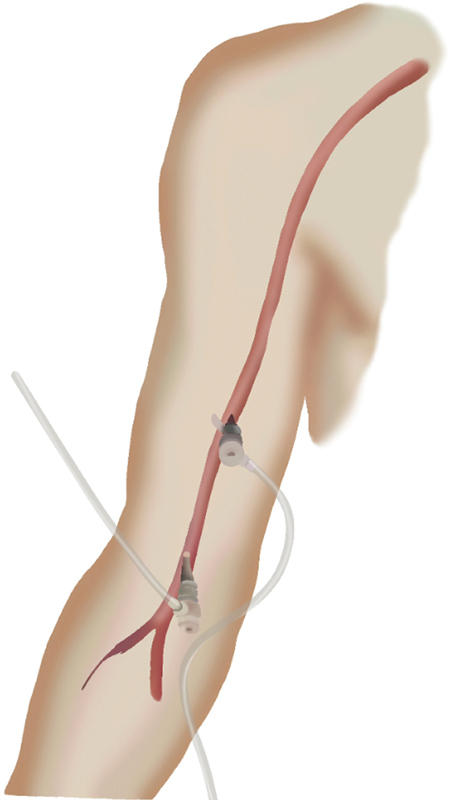
Artistic rendering of the double puncture in the right brachial artery. The first puncture is placed at the level of the medial epicondyle of the humerus, followed by a second puncture approximately 10 cm above the first.

In the current routine, using the Mandelli technique, in the case of four visceral branches, ultrasound-guided double puncture is used in the right and left brachial arteries ( Fig. 1 ). The first puncture is placed in the most distal segment (at the level of the medial epicondyle of the humerus, in the antecubital fossa), followed by a second puncture more proximally, approximately 10 cm above the first. For this second puncture, ultrasound or fluoroscopy can be used with the guidewire from the previous puncture as guide. Only one long sheath is passed into the brachial artery at a time. Based on our experience, we have a hematoma rate of 9% for proximal punctures and 4.5% at the epicondyle area. Our statistics indicate a 1.2% incidence of pseudoaneurysms, all of which were managed conservatively through Doppler-guided compression, without the need for surgical intervention.

After the main body of the endoprosthesis has been navigated via the femoral route to the healthy segment of the aorta immediately proximal to the aneurysmal disease, it is rotated to improve visualization of the fenestra and, then the endoprosthesis is released. Since we routinely use an endoprosthesis with a nitinol endoskeleton inside a polytetrafluoroethylene covering (APOLO; Nano Endoluminal), whenever we pass the wire through it, we advance the sheath with its introducer to confirm that there is no entanglement of the guidewire with the nitinol mesh. If the sheath advances easily, we are confident that no complications have occurred, and we proceed with the procedure. If the sheath catches on the nitinol, we remove it and repeat the step-by-step process for the proximal catheterization of the endoprosthesis. Next, a 5F/125-cm Vert catheter is positioned via the proximal brachial access. The arch is rotated for lateral visualization and the first target artery is catheterized, which may be the celiac trunk in the case of four branches or the superior mesenteric artery in the case of three branches. After confirming, by means of arteriography, that there was no dissection, the hydrophilic wire is replaced by an extra-stiff Amplatz 260 cm with a 1-cm tip. Thus, the long 6F × 90-cm sheath in the proximal brachial access is exchanged for a short 11-cm sheath and the long 6F × 90-cm sheath is reused in the distal brachial access for support and catheterization of the contralateral renal arteries.

**Fig. 2:**
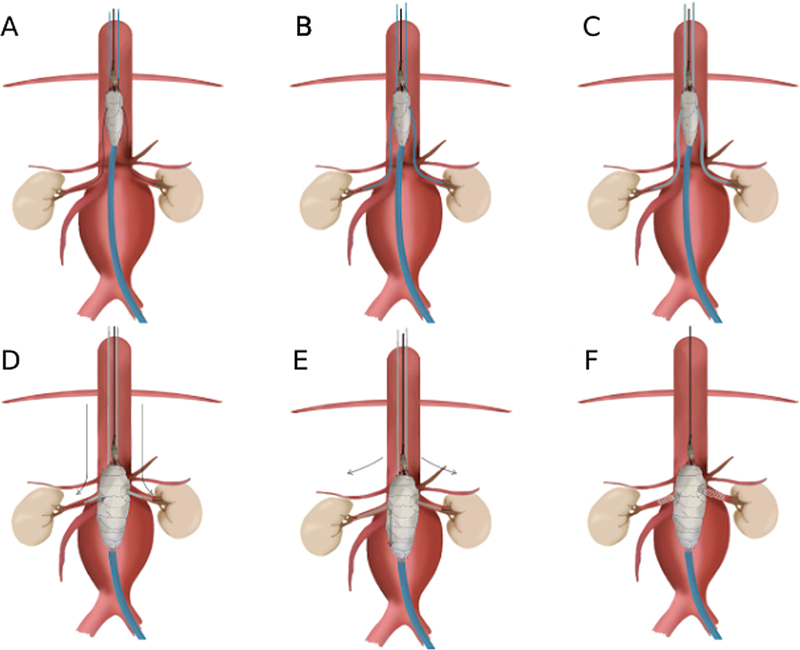
Artistic rendering of the complete procedure. After positioning the endograft in the healthy aorta to optimize the working space for catheterization of the target vessels. ( **A** ) The renal arteries were catheterized with a stiff guidewire. ( **B** ) The 5-F Vert catheter was advanced to the renal arteries with the goal of replacing the stiff guidewire with a super-stiff guidewire. ( **C** ) Proximal advancement of the introducer sheaths under the guidance of an Amplatz wire. Confirmation of the proper positioning of the sheaths in the renal arteries, without crossing them and without entangling the nitinol mesh. ( **D** ) Distal traction of the system was performed with rotation and sliding of the endograft along the sheath tracks until proper positioning was achieved. ( **E** ) Release of the diameter restrainers. ( **F** ) Implantation of the bridging stents.

**Fig. 3:**
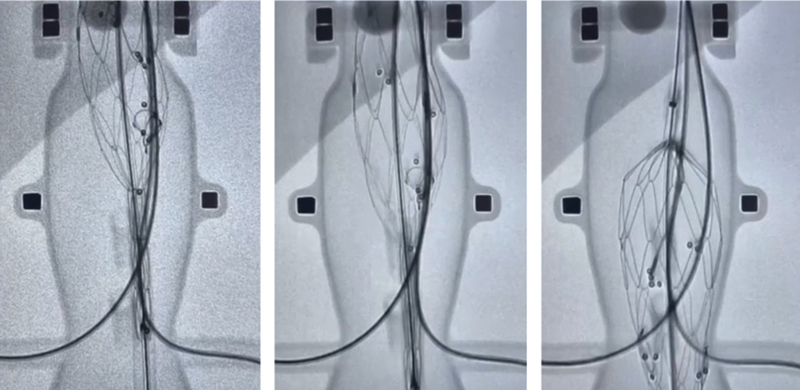
In vitro representation of the Mandelli sliding rail technique. The sequence of images allows us to observe the rotation of the endoprosthesis during sliding into the appropriate position.

**Fig. 4:**
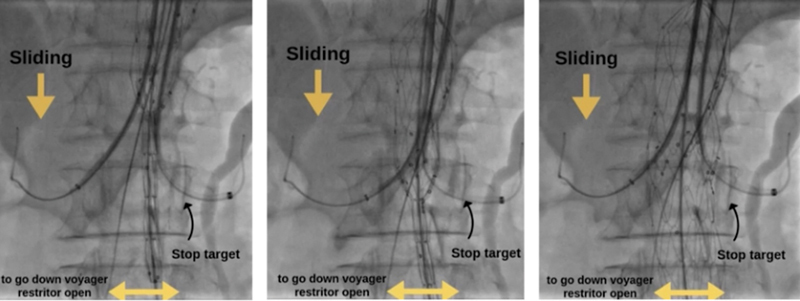
Radiographic representation of the Mandelli sliding rail technique. The image on the right shows us, after performing the maneuver, the release of the endoprosthesis diameter 60% restrictor.

Afterward, this entire process is repeated with catheterization of the renal arteries ( Fig. 2A, B ) and replacement of the hydrophilic guide with an extra-stiff Amplatz with a 1-cm tip. The sheath introducer is then inserted and positioned in the renal arteries, inside the fenestra ( Fig. 2C ). In this way, the sliding rails are made and the delivery device is pulled caudally until the endoprosthesis slides ( Fig. 2D ), settling into its ideal position ( Figs. 3 , 4 ) and, consequently, its complete release occurs with the removal of the restrictors ( Fig. 2E ). The most important point of attention is after the fenestration exit, as we need to ensure that, even if the endoprosthesis is rotated, the sheaths are not crossed. In other words, the sheath inserted through the right brachial artery must be catheterizing the right renal artery, and the sheath inserted through the left brachial artery must be in the left renal artery. This care ensures that, after distal traction with rotation to position the endoprosthesis correctly, the bridging stent will navigate smoothly along the previously created track.

**Fig. 5:**
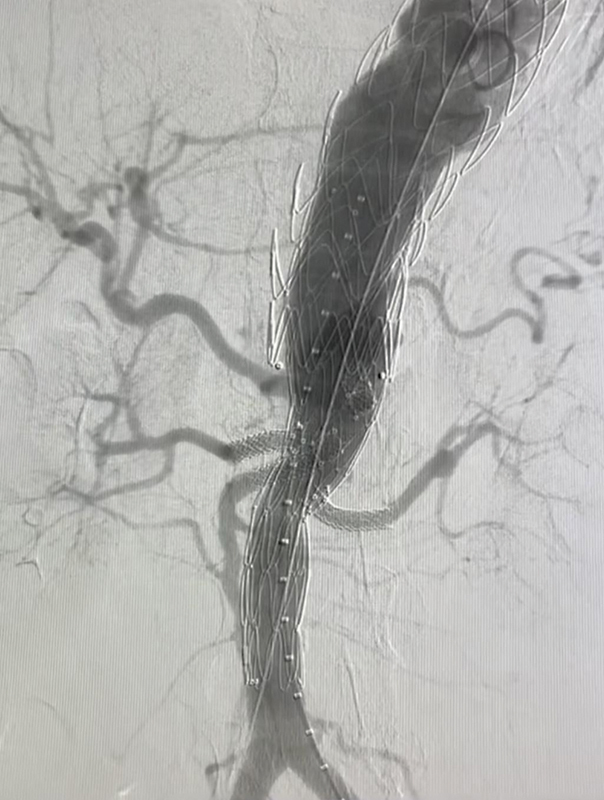
Postoperative aortography of the complete procedure. Implant the fenestrated visceral component inside the thoracic endoprosthesis implanted previously for the treatment of a thoracic aneurysm by sliding rail technique.

Next, the implantation of the bridging stents in the visceral branches is started. It is recommended to perform this procedure first in the renal arteries, due to the previous positioning of the sheaths ( Fig. 2F ). After the release of the stents, a new control angiography is performed to confirm the absence of dissections ( Fig. 5 ) and, only after this step, the sheaths can be removed and replaced by others in the guides in the superior mesenteric arteries and in the celiac trunk. The process is repeated and thus continues to the infrarenal correction.

## Discussion

Despite the variety of stents available for endovascular treatment of TAAAs, catheterization of visceral vessels in FEVAR can be technically challenging. This contributes to increased procedure duration, resulting in increased blood loss, elevated rates of limb ischemia, renal dysfunction, and reperfusion injury.

As is widely recognized, complex cases should be managed by experienced surgeons familiar with handling of specific stents. However, the integration of controlled release and repositionable devices with the Mandelli technique has revolutionized the approach by allowing precise repositioning and release of aortic stents. Consequently, this innovation has enabled significant advances in visceral artery catheterization techniques. Due to its ease of application by the surgeon and its anatomical suitability, the “Sliding Rail” technique offers a versatile option, which provides a more effective approach that is adaptable to the specific requirements of each case.

## Conclusion

The “Sliding Rail” technique, together with the advent of new recappable and repositionable endoprostheses, has proven to be an excellent alternative for endovascular repair of TAAAs, optimizing catheterization of visceral arteries. This approach adds to the range of options available to vascular surgeons in current clinical practice. In addition, the technique reduces procedure time and facilitates its reproduction, since it allows manipulation outside the target artery, even in situations involving significant angulation.
